# Temperature and chemical effects on the interfacial energy between a Ga–In–Sn eutectic liquid alloy and nanoscopic asperities

**DOI:** 10.3762/bjnano.13.72

**Published:** 2022-08-23

**Authors:** Yujin Han, Pierre-Marie Thebault, Corentin Audes, Xuelin Wang, Haiwoong Park, Jian-Zhong Jiang, Arnaud Caron

**Affiliations:** 1 KOREATECH – Korea University of Technology and Education, School of Energy, Material and Chemical Engineering, Cheonan, 31253 Republic of Korea; 2 UTT – University of Technology Troyes, 1004 Troyes, France; 3 International Center of New-Structured Materials (ICNSM), Laboratory of New-Structured Materials, State Key Laboratory of Silicon Materials, School of Materials Science and Engineering, Zhejiang University, Hangzhou, 310027, People’s Republic of China

**Keywords:** atomic force microscopy (AFM), interfacial energy, liquid alloy

## Abstract

The interfacial energies between a eutectic Ga–In–Sn liquid alloy and single nanoscopic asperities of SiO*_x_*, Au, and PtSi have been determined in the temperature range between room temperature and 90 °C by atomic force spectroscopy. For all asperities used here, we find that the interfacial tension of the eutectic Ga–In–Sn liquid alloy is smaller than its free surface energy by a factor of two (for SiO*_x_*) to eight (for PtSi). Any significant oxide growth upon heating studied was not detected here, and the measured interfacial energies strongly depend on the chemistry of the asperities. We also observe a weak increase of the interfacial energy as a function of the temperature, which can be explained by the reactivity between SiO*_x_* and Ga and the occurrence of chemical segregation at the liquid alloy surface.

## Introduction

Recently, room-temperature-liquid Ga-based alloys have been attracting interest from various scientific communities, including chemical [1], biomimetic [2], microfluidic [3], electrical [4], and materials science [5]. This increased interest is due to the low viscosity and high and electrical conductivity of these alloys, on the one hand, and their non-toxicity and low vapor pressure, on the other hand. Room-temperature-liquid Ga-based alloys are considered for direct writing and printing stretchable and flexible electronic devices, such as antennas or wires [5–7]. Such applications and the related processing of liquid metals strongly depend on their surface and interfacial properties.

The surface tension of room-temperature-liquid Ga-based alloys has been reported to be lowered by a thin surface oxide layer [8]. In [9], the authors electrochemically controlled the growth and removal of gallium oxide to tune the surface tension of liquid gallium. Oxygen is a surface-active substance whose effect on surface tension has been investigated for various liquid metals [10]. In metallic alloys, surface segregation has also been observed, where an element with a higher oxygen affinity enriches at the surface to form an oxide [10]. This effect has also been used to trigger the reaction of thin oxide films at the liquid–vapor interface with liquid gallium alloys [11]. While the liquid–vapor interface of liquid gallium-based alloys has been well investigated, the wetting of liquid gallium alloys on different substrates has not yet attracted as much attention. Recently, the authors of [12] highlighted the role of the oxide skin on the adhesion strength of gallium-based alloys on various substrates. Specifically, the authors found that the resulting adhesion strength is low when the oxide skin surrounding a liquid drop is not disrupted during application onto a substrate.

In contrast, when the oxide skin breaks, new oxide forms at the solid–liquid interface with a substrate, which results in adhesion. Also, the wetting of a liquid Ga–In alloy has been related to the adsorption energy of gallium on three different substrates (steel, gold, and Al) [13], with the wetting becoming better as the adsorption energy of gallium onto the substrate becomes more negative. In the case of Fe and Cu substrates, it was observed that liquid gallium reacts with the substrate to form an intermetallic layer at the gallium–substrate interfaces, which promotes the wetting of the gallium melt [14–15]. Similarly, room-temperature-liquid eutectic Ga–In and eutectic Ga–In–Sn alloys have been reported to reactively wet thin indium and tin foils [16]. Also in [16], the authors demonstrated that the wetting of the same liquid alloys could be tuned by texturing the substrate surface.

The wetting of gallium-based liquid alloys is thus complex and depends on the stability of the oxide at the liquid–substrate interface, the reactivity with the substrate material, and the substrate topography. In this work, we applied atomic force spectroscopy to determine the interfacial energy between eutectic Ga–In–Sn liquid alloy and single nanoscopic asperities of SiO*_x_*, Au, and PtSi in the temperature range between room temperature and 90 °C. The choice of the asperity materials was motivated by their relevance in electronics and micro-/nanotechnology. The surface chemical composition of the liquid alloy was measured by X-ray photoelectric spectroscopy before and after heating to 100 °C for 3 h. Furthermore, we imaged the nanoscopic asperities after measurements on the metallic liquid alloy by SEM to evidence possible liquid residues. For all asperities used in this work, we find that the interfacial tension of the eutectic Ga–In–Sn liquid alloy is smaller than its free surface energy by a factor of two (for SiO*_x_*) to eight (for PtSi). While we did not observe any significant oxide growth upon heating, the measured interfacial energies strongly depend on the chemistry of the asperities. Furthermore, we observe a weak increase of the interfacial energy as a function of the temperature. We discuss our results based on the reactivity between SiO*_x_* and Ga and the occurrence of chemical segregation at the liquid alloy surface.

## Experimental

We prepared a eutectic Ga–In–Sn liquid alloy by melting the mixture of its solid constituents with the composition of 78.8 atom % Ga, 13.2 atom % In, and 8 atom % Sn. The melting point of the alloy is approximately 283 K [17]. We measured the interfacial tension between the Ga–In–Sn liquid eutectic alloy and atomic force microscopy (AFM) tips of different chemistries as a function of the temperature (*T* = 21–90 °C) by AFM force spectroscopy using an XE100 AFM equipped with a heating stage (manufactured by Park Instruments, Republic of Korea). We recorded force–distance curves with PtSi-coated Si cantilevers (PtSi-cont, manufactured from NanoSensors, Switzerland), SiO*_x_* cantilevers (Contsc, manufactured from NanoSensors, Switzerland), and Au-coated Si cantilevers (ContscAu, manufactured from NanoSensors, Switzerland). Before measurements, the sensitivity of the AFM photodiode was calibrated by recording a force–distance curve with each cantilever on a quartz glass sample (manufactured by Goodfellow, United Kingdom) and extracting its slope in the range of repulsive forces. Subsequently, we determined the bending stiffness *C_n_* of each cantilever by analyzing its thermal noise vibration [18]. After measurements, each tip was investigated by SEM for possible material transfer from the liquid alloy sample. The values of the half-opening angle θ of the tips were taken from the manufacturer’s data. Table 1 summarizes the properties of the cantilevers used in this work.

**Table 1:** Cantilever properties.

	PtSi-Cont	Contsc	ContscAu

*C_n_* [N/m]	0.24	1.14	0.89
θ [°]	12.5	5	12.5

**Figure 1:**
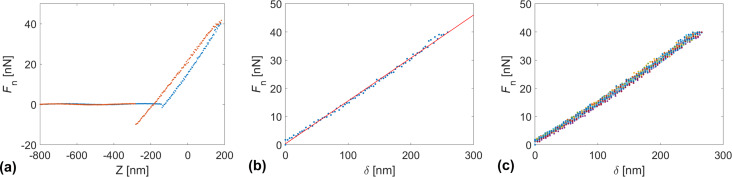
(a) Typical force–distance *F*_n_(*Z*) curve recorded with a SiO*_x_* tip on the eutectic Ga–In–Sn melt at room temperature; the blue markers indicate the approach part, while the orange markers indicate the retraction part. (b) Force–penetration *F*_n_(δ) curve, calculated from the approach part of the *F*_n_(*Z*) curve in (a) and its corresponding fit in red. (c) Superposition of 15 *F*_n_(δ) curves recorded under the same conditions, that is, temperature and approach velocity.

The force spectroscopy measurements consisted of approaching a cantilever towards the sample surface at varying velocities d*Z*/d*t* = 0.1–25 µm/s (see Figure 1). We repeated force spectroscopy measurements at each approach/retraction velocity 15 times.

We used the retraction part of the force–distance curves to determine the adhesion force *F*_ad_ and calculate the corresponding work of adhesion *W*_ad_ as suggested in [19] for solid interfaces. The authors measured the adhesion between atomically smooth quasicrystalline surfaces of TiN-coated AFM tips in ultrahigh vacuum by analyzing the pull-off force during atomic force spectroscopy measurements. The authors compared different modifications of the Hertzian contact mechanical model for elastic solids to account for adhesion. The authors found that the measured adhesion significantly depends on the maximum load applied before retraction. In the case of a plastic contact, adhesion was larger than for an elastic contact, owing to the formation and rupture of chemical bonds between tip and sample in the former case. Furthermore, we used the approach part of the curves to calculate the force–penetration curves according to δ = *Z* − *F*_n_/*C*_n_. The determination of the interfacial energy γ between an AFM tip and a metallic liquid alloy is based on a balance between the pressure applied by the tip onto the liquid surface and the restoring pressure due to the line tension at the liquid interface, that is, *p*(δ) = *F*_n_(δ)/*A*(δ) = γ/*P*(δ), where *A*(δ) is the contact area and *P*(δ) is the perimeter between tip and liquid. Assuming a conical shape for an AFM tip, the perimeter *P*(δ) and the contact area *A*(δ) can be expressed as *P*(δ) = 2πδ·tan θ and 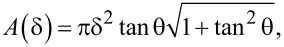 where θ is the half-opening angle of the tip, which we determined by scanning electron microscopy. We determined the interfacial energy γ between tip and liquid sample by fitting the function


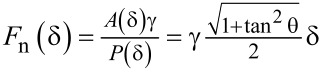


to our experimental data.

The chemical composition of the eutectic Ga–In–Sn liquid alloy sample was determined by photoelectronic X-ray spectroscopy (XPS) before and after heating in air at 100 °C for 3 h. The spectrograms were recorded with a K-alpha^+^ XPS system manufactured by ThermoFischer Scientific, USA. We used a monochromated Al Kα source and a spot size of 400 µm. The results presented below consist of the average of ten consecutively recorded measurements.

## Results and Discussion

**Figure 2:**
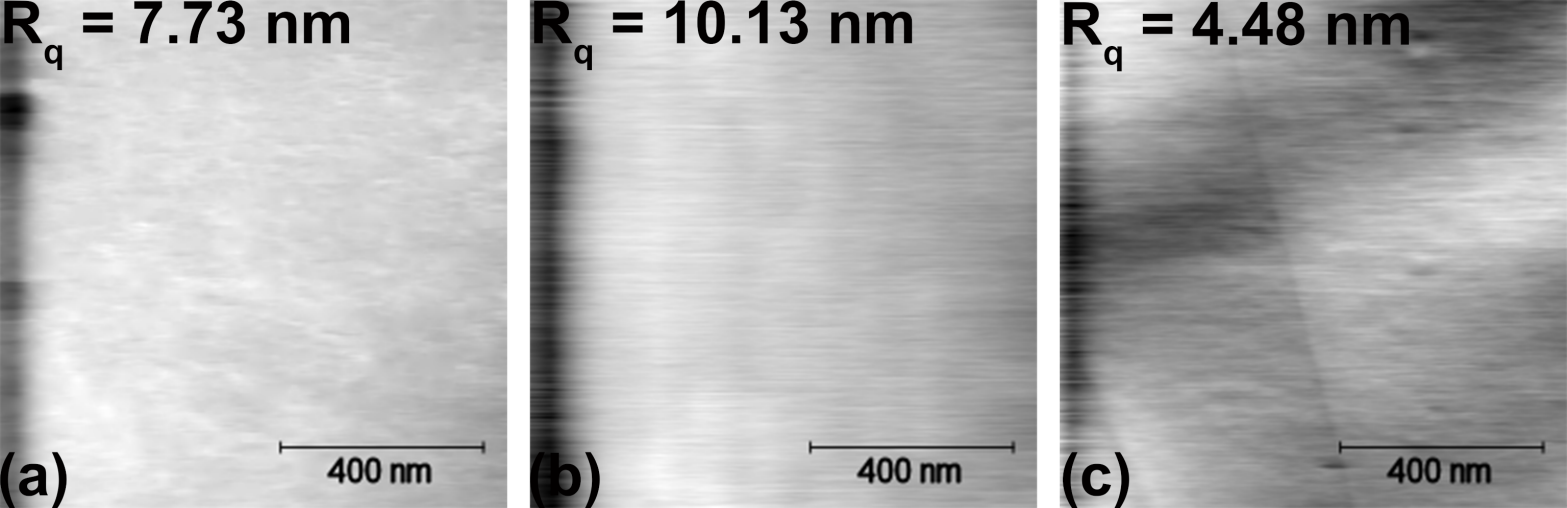
Contact AFM topography images recorded on the surface of eutectic Ga–In–Sn with (a) SiO*_x_*, (b) PtSi, and (c) Au AFM tips. We set the normal force to *F*_n_ = 2 nN and the scanning velocity to *v*_s_ = 4 µm/s.

Figure 2 shows contact AFM topography images recorded at room temperature on the surface of the eutectic Ga–In–Sn melt with three AFM tips of different chemistries, namely SiO*_x_*, PtSi, and Au. Figure 2 also indicates the surface roughness *R*_q_ value for each topography image. We recorded the presented topography images in contact mode by setting and controlling the normal force to *F*_n_ = 2 nN and the sliding velocity to *v*_s_ = 4 µm/s. Depending on the tip chemistry, we determined different roughness values ranging from *R*_q_ = 4.48 nm with a gold tip to *R*_q_ = 10.13 nm with a platinum silicide tip. This dependence on the tip chemistry likely originates from different capillary interactions between the eutectic Ga–In–Sn melt and the tips, where large capillary forces would result in a larger apparent topography.

**Figure 3:**
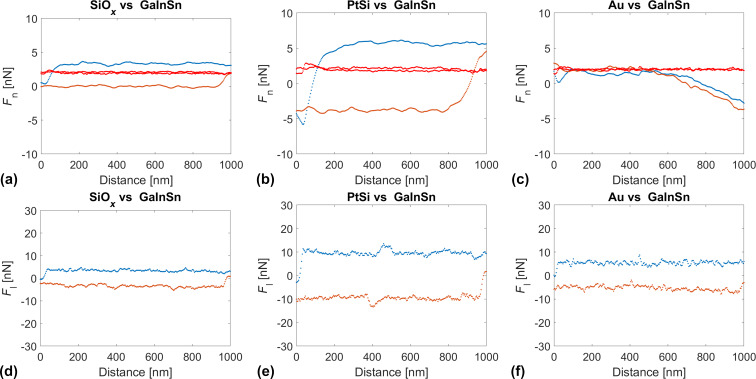
Typical (a–c) *F*_n_- and (d–f) *F*_l_-traces recorded in contact AFM mode on the surface of the eutectic Ga–In–Sn melt with (a, d) a SiO*_x_* tip, (b, e) a PtSi tip, and (c, f) an Au tip.

We can infer the occurrence of capillary interactions during the sliding of an AFM tip on the surface of the eutectic Ga–In–Sn melt from the traces of the normal and lateral forces plotted for both forward and backward directions as presented in Figure 3. There, the normal force plots show the normal force signal as controlled by the feedback loop in red color, while the normal force traces in blue and orange colors were calculated from the height traces in, respectively, the forward and backward direction by multiplying the height signal with the bending stiffness. We observe a hysteresis for both normal and lateral forces, which indicates a dragging force opposing the sliding motion of the tip. The observation of hysteresis for the lateral force is common on solid surfaces and corresponds to friction. In our case, we attribute the observed hysteresis of the lateral force to the resistance to pull and drag a meniscus at the tip–metallic liquid interface. In principle, this resistance corresponds to the interfacial tension of the tip and metallic liquid. The occurrence of hysteresis for the normal force is not as straightforward to explain. In our experiments, we can exclude the effect of crosstalk due to misalignment of the cantilever since we mounted each cantilever on the same alignment chip on the cantilever holder. We consider it more likely that the hysteresis of the normal force arises from a tilt between cantilever and the liquid surface.

**Figure 4:**
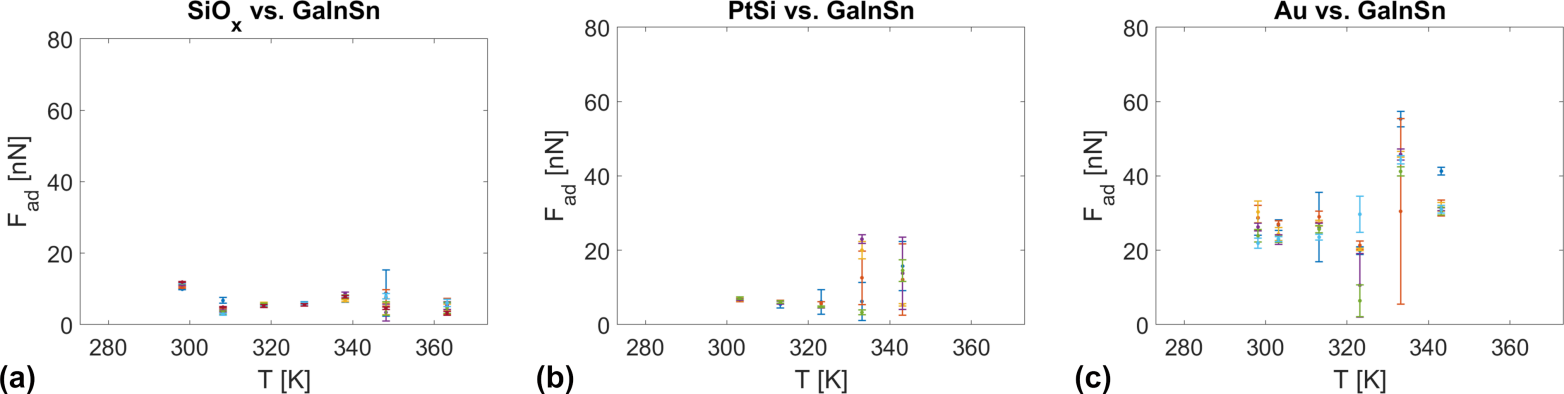
*F*_ad_(*T*) plots for different pulling velocity values d*Z*/d*t* = 0.25–25 mm/s for (a) a SiO*_x_* tip, (b) a PtSi tip, and (c) a gold tip.

Figure 3 shows that the magnitude of the dragging force, that is, the sum of half-widths of the normal and lateral force hystereses, strongly depends on the tip chemistry. It is maximal for the PtSi tip and minimal for the Au tip. These preliminary results indicate that adhesion and capillary effects affect the tip–liquid contact. In particular, Figure 2 and Figure 3 indicate that the contact between SiO*_x_* and PtSi is rather inelastic. In a first attempt to characterize the interfacial tension between the surface of the eutectic Ga–In–Sn melt, we determined the adhesion force between the eutectic Ga–In–Sn melt and AFM tips of the abovementioned chemistry as a function of the temperature and the pulling velocity. Figure 4 summarizes these results. We do not observe any clear tendency for the adhesion force regarding temperature or pulling velocity for all presented cases. However, by taking the average of all adhesion force values for each tip, we find the following mean values and associated standard deviations: 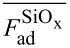 = 6.46 ± 2.35 nN, 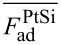 = 7.77 ± 4.61 nN, and 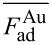 = 29.19 ± 9.71 nN. Adhesion of a gold tip on the eutectic Ga–In–Sn melt is significantly larger than for SiO*_x_* and PtSi tips, which makes a clear distinction difficult. It is, however, noteworthy that the largest adhesion corresponds to the lowest dragging force, as illustrated in Figure 3. Above, we already discussed the meniscus formation at the tip–liquid interface. Here, we attempt to estimate the work of adhesion from the adhesion force according to the Johnson–Kendall–Roberts (JKR) model of adhesive contact [20], where *W*_ad_ = 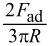 and *R* is the tip radius. Strictly, this model applies to adhesive contact between elastic solids that form a wetting neck. Contact between two elastic solids is not provided in our experiments, and liquid flow will likely alter the calculated work of adhesion values. These values should thus be taken as rough estimates. With the manufacturer’s data for the tip radius (*R*_SiO_*_x_* = 7 nm and *R*_PtSi_ = *R*_Au_ = 25 nm), we calculate the following work of adhesion values: 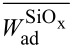 = 195.83 ± 71.24 mN/m, 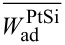 = 65.95 ± 3.79 mN/m, and 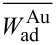 = 247.76 ± 82.42 mN/m. Furthermore, the work of adhesion can be expressed according to 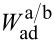 = γ_a_ + γ_b_ + γ_a/b_, where γ_a/b_ is the surface energy of the bodies a and b, and γ_a/b_ is the interfacial energy between the bodies a and b. The surface energy of the eutectic Ga–In–Sn melt at the melting point has been reported to be 587 mN/m [21], while published values for SiO*_x_*, PtSi, and Au are 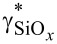 = 53 mN/m [22], 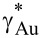 = 1150–1300 mN/m depending on its orientation [23], 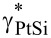 = 1710 mN/m [24]. These values lead to the following results for the interfacial energy values of the different couples investigated in this work: 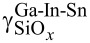 = 0.44 N/m, 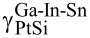 = 2.2 N/m, 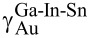 ≈ 1.5 N/m. These values are, in part, significantly larger than the reported surface energy value of the eutectic Ga–In–Sn melt. Besides the limitation of the JKR model to describe our experiment, due to the meniscus flow of the eutectic Ga–In–Sn melt upon retraction, the adsorption of a thin water layer is likely to modify the surface energy of the liquid neck upon pulling and affect the above results. The analysis of the interfacial energy from AFM adhesion measurements is thus complicated by the absence of a suitable model and environmental effects.

**Figure 5:**
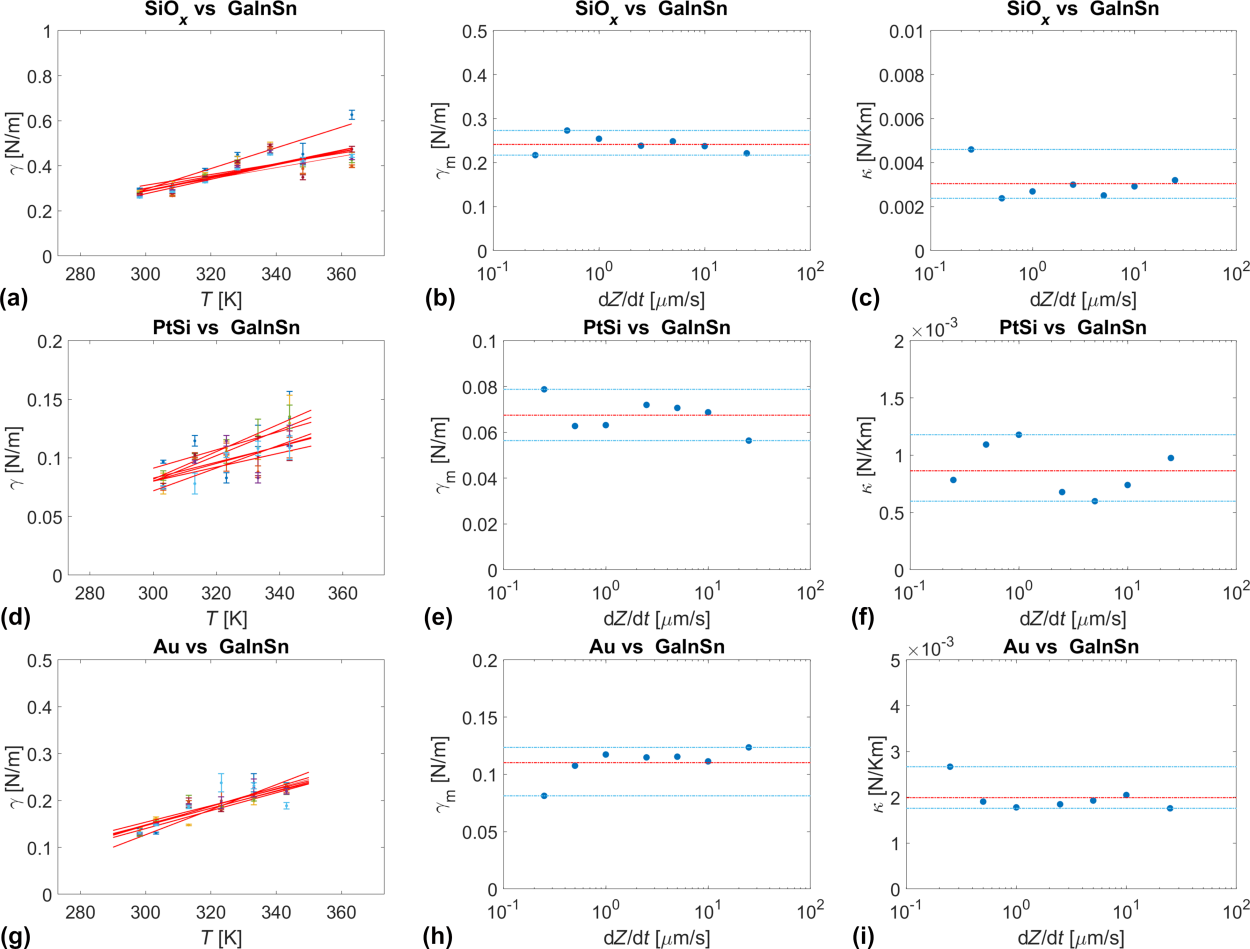
(a, d, g) Interfacial energy γ as a function of the temperature determined with d*Z*/d*t* = 0.25–25 mm/s; (b, e, h) interfacial energy at the melting point γ_m_ as a function of d*Z*/d*t* (the mean value of γ_m_ is indicated as a red dashed line and the range of values is indicated by two blue dashed lines); (c, f, i) temperature sensitivity κ of the interfacial energy as a function of d*Z*/d*t* (the mean value of κ is indicated as a red dashed line and the range of values is indicated by two blue dashed lines).

Alternatively, we determined the interfacial tension between AFM tips and the eutectic Ga–In–Sn melt from the approach part of force–distance curves recorded at different temperatures and approaching velocities. In this case, the tips penetrated the liquid sample to depths of several hundreds of nanometers. For this reason, we can neglect the effect of adsorbed water and assume an intimate contact between the tip material and the eutectic Ga–In–Sn melt. Also, unlike in adhesion experiments, where a pulled neck changes the geometry of the tip–liquid interface and may involve a flow of the liquid sample, the tip–liquid interface geometry is better defined upon penetration of the tip in the liquid. Figure 5 shows the temperature and velocity dependences of the interfacial energy between eutectic Ga–In–Sn melt and three AFM tips of SiO*_x_*, PtSi, and Au. For each d*Z*/d*t*-values, the temperature dependence of γ was fitted with the linear function γ(*T*) = γ_m_ + κ(*T* – *T*_m_), where γ_m_ is interfacial at the melting point, κ is the temperature sensitivity of γ, and *T*_m_ is the melting point of eutectic Ga–In–Sn melt. The values of γ_m_ and κ are also shown as a function of d*Z*/d*t* in Figure 5. For all three tips, we observe slight increases in γ with the temperature. γ(*T*) significantly depends on the tip chemistry. γ_m_ and κ are lowest for PtSi tips and increase in the order of Au tip and SiO*_x_* tip. However, these values do not appear to depend on the approach velocity of the tips toward the liquid sample. Table 2 indicates the average values and ranges of γ_m_ and κ for the three different tip chemistries. Specifically, we find 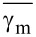 = 230 mN/m and 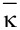 = 3 mN/Km for SiO*_x_*, 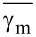 = 110 mN/m and 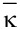 = 2 mN/Km for Au, and 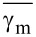 = 68 mN/m and 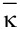 = 0.8 mN/Km for PtSi.

**Table 2:** Average values and range of γ and κ for PtSi, SiO*_x_*, and Au tip materials.

	PtSi	SiO*_x_*	Au

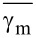 [mN/m]	68	230	110
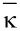 [mN/Km]	0.8	3	2

The surface tension of Ga–In–Sn eutectic liquid alloy at the melting point has been reported to be 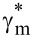 = 587 mN/m, while its temperature sensitivity is κ* = −10 mN/Km [21]. This value for the surface energy is expected to be larger than the interfacial energy values determined in this work. However, the positive κ values in this work are unexpected and require a thorough discussion. In the following, we also discuss the effect of tip chemistry and oxide growth on the interfacial energy of Ga–In–Sn.

**Figure 6:**
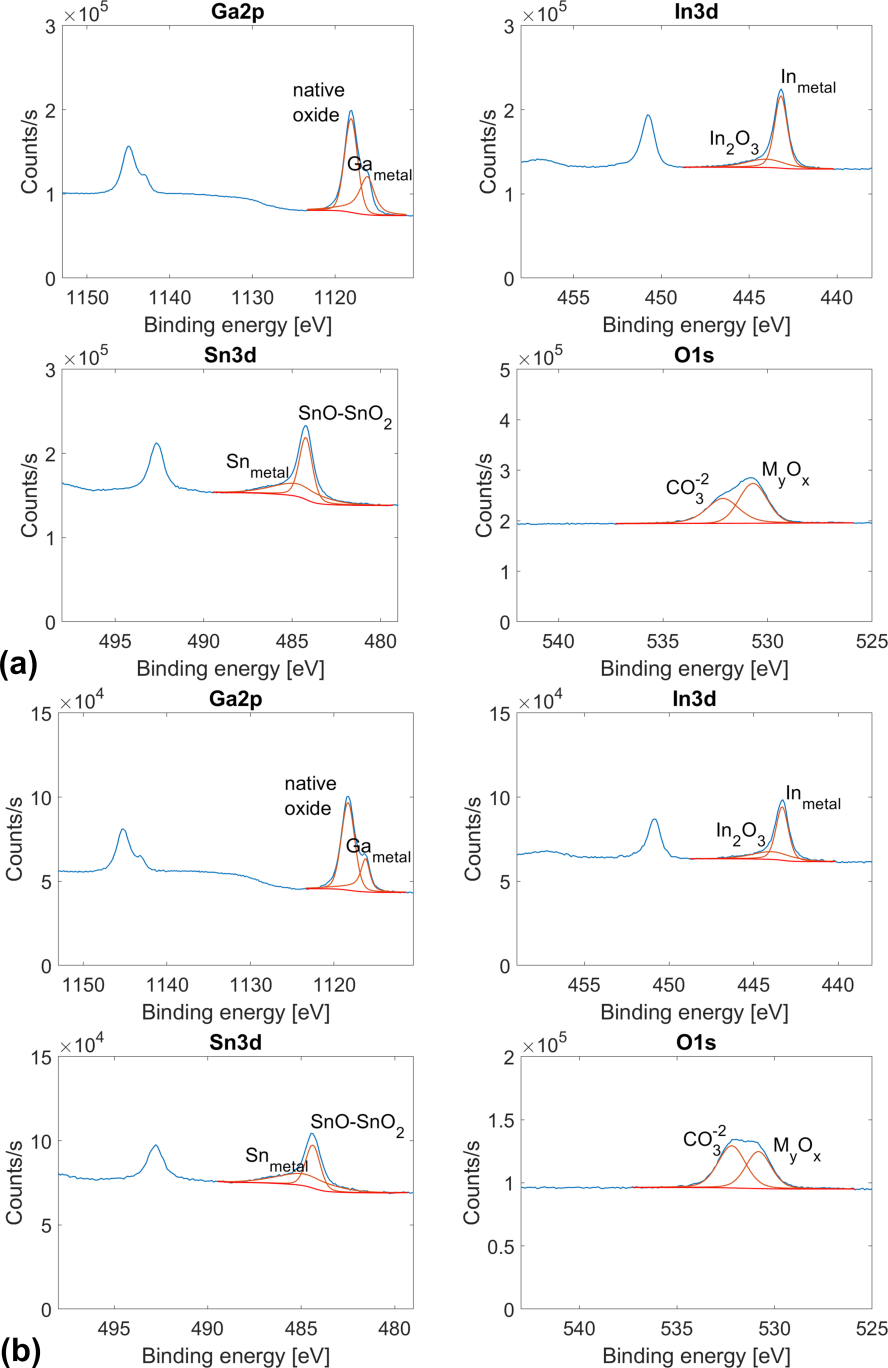
XPS results for the eutectic Ga–In–Sn melt (a) before and (b) after heating at 100 °C for 3 h.

The measurements presented here were performed in air. Thus, a thin native oxide layer, which may have grown upon heating, likely affected our measurements. Figure 6 and Table 3 show XPS results obtained on a drop of Ga–In–Sn eutectic liquid before and after heating in air at 100 °C for 3 h. The choice of this duration roughly corresponds to the time necessary to complete a series of AFM measurements.

**Table 3:** Surface chemical composition of Ga–In–Sn eutectic melt (a) before and (b) after heating for 3 h at 100 °C.

	Ga_metal_ [atom %]	Ga_oxide_ [atom %]	In_metal_ [atom %]	In_In2O3_ [atom %]	Sn_metal_ [atom %]	Sn_SnO-SnO2_ [atom %]	O_MyOx_ [atom %]

as received	9.7	23.2	4.9	1.7	2.9	3.9	53.7
after heating at 100 °C	10.2	27.0	4.4	2.0	3.3	3.2	49.9

Figure 6 and Table 3 show that, after heating, the oxygen concentration at the surface of the Ga–In–Sn eutectic liquid slightly decreased. Note that the 1s orbital of oxygen indicates the presence of carbonate groups at the surface of the Ga–In–Sn eutectic melt. Similarly, and not shown here, we observed a signal corresponding to the 1s orbital of carbon. We attribute these contributions (CO_3_^2−^, C–C/C–H, and C–O) to contamination from the ambient. As mentioned above, we performed the XPS measurements on these liquid samples without prior Ar^+^-ion sputtering or further heating inside the vacuum chamber of the XPS instrument.

Given that these contributions arose from contamination by the ambient, we excluded them from our calculations of the surface chemical composition. Figure 6 shows that the surface oxide on the melt mainly consists of gallium and tin oxides, with a minor contribution from indium oxide. After heating at 100 °C for 3 h, this general trend was preserved.

Table 3 shows that after heating, the oxygen concentration at the surface of Ga–In–Sn eutectic liquid slightly decreased, that is, from 53.7 to 49.9 atom %. Moreover, the atomic fraction of gallium bound as native oxide increased from 23.2 to 27.0 atom %, while the atomic fractions of tin and indium bound as oxides (SnO or SnO_2_, and In_2_O_3_) did not significantly change. Given the atomic concentrations in Table 3, the native mixed surface oxide of the Ga–In–Sn eutectic liquid consists of GaO_2_–Sn_2_O_3_–In_2_O_3_ or (Ga_0.8_Sn_0.14_In_0.06_)O_2_. After heating, this concentration changed to (Ga_0.84_Sn_0.10_In_0.06_)O_2_. The enrichment of the surface oxide in gallium at the expense of oxygen may indicate that oxygen was brought into solution in the Ga–In–Sn bulk liquid during heating.

Further, we estimate the change of the surface oxide thickness based on the change of the ratio between Ga_native oxide_ and Ga_metal_. Before heating, we calculated this ratio to be 2.38, while after heating, we found 2.64. The slight increase in the Ga_native oxide_/Ga_metal_ ratio after heating indicates that the surface oxide thickness did not significantly increase if we consider the oxide enrichment in Ga. According to the manufacturer, the interaction depth of the XPS was approx. 10 nm. We detected metallic bonded Ga, In, and Sn; thus, we infer that the oxide layer was less than 10 nm thick. Thickness and composition of the surface oxide of a similar newly developed Ga–In–Sn-Zn liquid alloy have been characterized by TEM and XPS [25]. The authors reported on the effect of electron beam exposition time on the growth of a ZnGa_2_O_4_ layer. After 35 min irradiation, the oxide layer had grown from 2 nm to less than 5 nm. Besides this moderate growth, the authors observed the partial crystallization of the oxide layer during electron beam exposition. We measured the temperature dependence of the interfacial energy between Ga–In–Sn eutectic liquid and AFM tips by dipping the AFM tips inside a Ga–In–Sn liquid drop. The penetration depth of the tips was in the order of several hundreds of nanometers.

We did not observe any pop-in in the force–penetration curves that would indicate a sharp rupture of the oxide film. Such an observation required a higher sampling rate than used during the measurements. However, the experimental data points deviate from the linear fit function at penetration depth values below 50 nm (see Figure 4b,c). At low penetration depth values, the contact between tip and liquid may not be intimate yet, and the observed deviation from linearity in the *F*_n_(δ) plots may correspond to a pre-tension effect of the interface. The penetration of the tip at larger depths is thus expected to be representative of the contact between nanoscopic asperities and metallic melt.

Furthermore, we estimated the chemical composition of the metallic melt below the oxide layer from the concentrations listed in Table 3. In the case of as-received Ga–In–Sn eutectic liquid, we find Ga_55.38_In_28_Sn_16.62_, while after heating, we find Ga_56.75_In_24.61_Sn_18.64_. Both compositions strongly deviate from the nominal composition of the alloy Ga_78.8_In_13.2_Sn_8_. These results hint at chemical segregations at the metallic liquid surfaces, whose net effect is reducing the surface tension of the liquid alloy (see discussion below).

To discuss the effect of tip chemistry and temperature on our results, it shall be convenient to remind the reader about the physical origin and the thermodynamic interpretation of surface and interfacial energies. The surface tension arises from the imbalanced bonding of atoms at the liquid–vapor interface. Interfacial atoms are attracted by atoms in the bulk liquid to experience a pulling force in the direction from the surface to the bulk of the liquid. This effectively results in the tendency of the system to minimize its interfacial area with vapor or a vacuum. Hence, the surface tension can be defined as the work to create a new unit area of surface reversibly. For a single component liquid, this translates as γ*A = *F*_s_, where *A* is the surface area and *F*_s_ is the Helmholtz free energy of the surface. In the case of a multicomponent and single-phase liquid, this equality is reduced by chemical segregation at the surface, that is, 
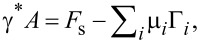
 where µ*_i_* is the chemical potential of the *i-*th component and Γ*_i_* is its adsorption [26]. By neglecting the effect of surface segregation on the surface tension, the differential of the Helmholtz free energy of the surface can be written as d*F*_s_ = γ*d*A* + *A*dγ* or 
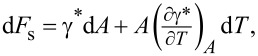
 which allows for a correlation of the thermal sensitivity of the surface or interfacial tension 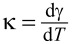 with the surface or interfacial entropy 
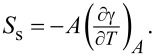
 According to [27–28], the temperature sensitivity of the surface energy of simple metallic melts depends on the change of the cohesion energy with temperature and, thus, scales with their molar area and thermal expansion coefficient.

**Figure 7:**
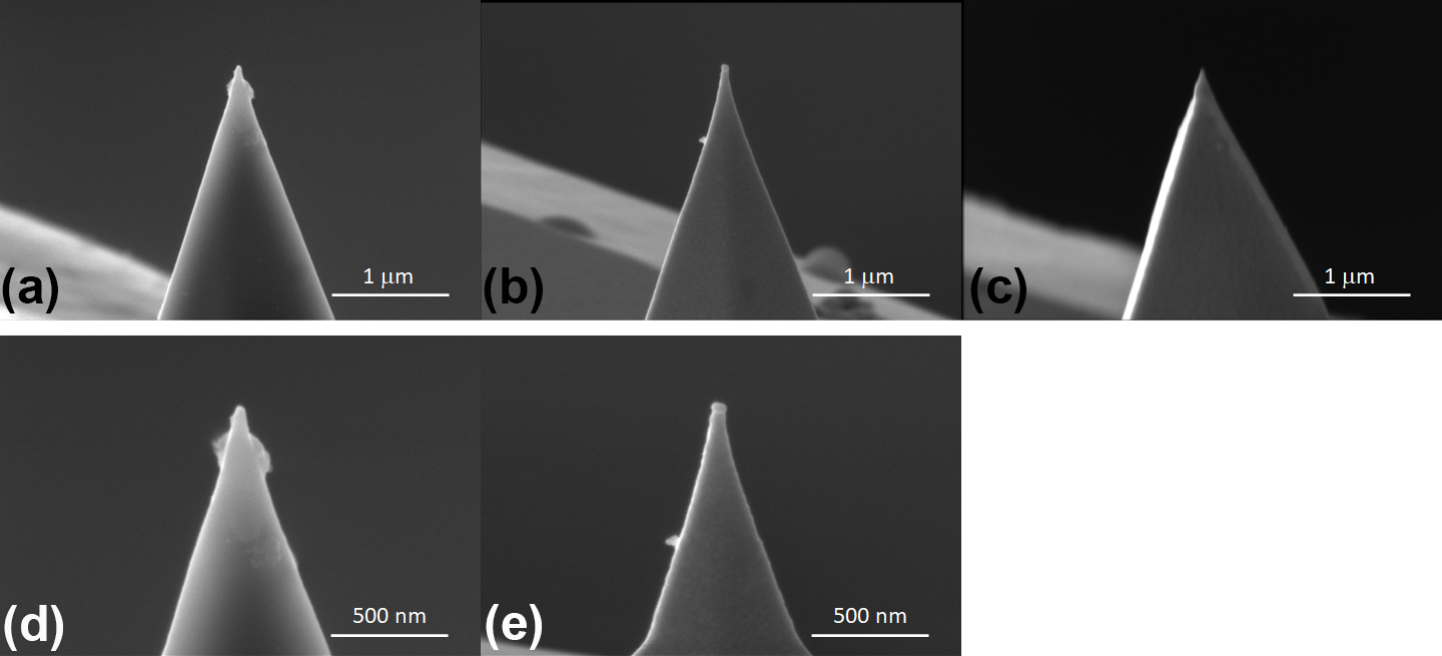
SEM images of the AFM tips used to collect the results presented in Figure 2: (a, d) SiO*_x_* tip, (b, e) Au tip, and (c) PtSi tip.

The surface tension of the eutectic Ga–In–Sn liquid at the melting point has been reported to 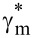 = 587 mN/m. Since the surface tension originates from the imbalanced bonding of atoms at the liquid–vapor interface [29], it shall be no surprise that the interfacial tension of the same liquid in contact with a solid is smaller since a solid surface also consists of atoms with unsaturated bonds that can minimize their energy by bonding with atoms from the liquid surface. Depending on the chemistry of the AFM tip, we find that a factor of two to eight reduces the interfacial energy compared to the surface energy. Figure 7 shows SEM images of the tips after measurements on the Ga–In–Sn eutectic liquid. Unlike the PtSi and Au tips, the SiO*_x_* tip in Figure 7 exhibits residues of the liquid alloy up to a height from the tip apex of *h* ≈ 200 nm, which corresponds to the penetration depth of the tip into the liquid alloy. Coincidently, we determined the largest interfacial tension value at the melting point of the liquid alloy for the same tip, 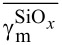 = 230 mN/m. The adhesion of melt residues at the SiO*_x_* tip can be attributed to the respective stabilities of the oxides at the tip and at the liquid surface, respectively. Their stability can be discussed based on their respective melting points and enthalpies of fusion Δ*H*_fus_ and formation at *T* = 298.15 K, 
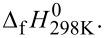
 For amorphous SiO_2_, the following values were reported: 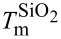 = 1726 K, 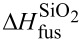 = 7.438 kJ/mol, and 
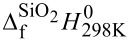
 = −910.68 kJ/mol [30]. For Ga_2_O_3_, the literature reports 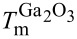 = 2080 K, 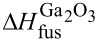 = 99.77 kJ/mol, and 
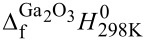
 = −1090.85 kJ/mol [30]. Hence, it appears that Ga_2_O_3_ is significantly more stable than SiO_2_, and we suggest that upon penetrating the Ga–In–Sn eutectic melt, oxygen atoms at the tip surface react with Ga to form a solid Ga_2_O_3_ layer at the tip–melt interface. In this scope, the 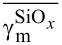 value determined in this work may have been increased by the presence of a solid Ga_2_O_3_ layer at the tip–melt interface. Our interpretation is supported by recent calculations of the surface energy of Ga_2_O_3_ in [31]. Depending on the surface orientation, a range of values between 0.6 N/m and 2.98 N/m has been reported.

The values of 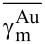 and 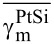 determined in this work are significantly lower than 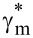 and 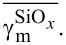 Due to its chemical inertness, reactivity between PtSi and eutectic Ga–In–Sn melt can be ruled out. Also, reactions between gold and the constituting elements of the metallic melt are not expected. Solid gallium has very poor solubility for gold, and Ga forms a eutectic with AuGa_2_ with a melting temperature of 491 °C. We thus exclude its formation during our experiment. However, the Ga-rich melt has a large solubility for Au.

For this reason, we assume that a small amount of gold dissolved in the metallic melt investigated here. However, the dissolution of Au should be small since it is thermally activated. In the current study, we could not observe any degradation of the Au tip nor measure traces of Au in liquid Ga–In–Sn. The phase diagram of the Au–In alloy system is similar to that of Au–Ga. However, the difference is that the eutectic formed between In and AuIn_2_ lays at 156 °C, higher than the maximum temperature applied during our measurements. Finally, the Au–Sn system shows no solubility of Au in Sn and exhibits a eutectic between AuSn_4_ and Sn at 211 °C. A reaction between Au and Sn can thus be ruled out for our experimental conditions.

In [23], the authors calculated the surface energy of gold nanoparticles of different shapes. The authors found that the surface energy sharply increases for diameters smaller than 5 nm for spherical particles. In our experiments, the Au tip apex can be considered a sphere with a radius *R* = 25 nm. According to [23], the surface energy of the gold tip can be assumed to be in the range of 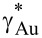 = 1.15–1.30 J/m^2^. The surface energy of stochiometric PtSi(010) was calculated as a function of the number of (010) planes below the surface [24]. A slight decrease in surface energy was observed upon increasing the number of planes. For a supercell consisting of 25 (010) planes, the authors in [24] calculated the surface energy of PtSi, 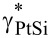 = 1.71 J/m^2^. It is thus remarkable that the higher the surface energy of the tip material, the lower the interfacial energy between the tip and the Ga–In–Sn eutectic melt becomes. This result is so far expected as forming a tip–liquid interface rids areal parts of the tip–vapor and the Ga–In–Sn eutectic melt–vapor interfaces and, thus, decreases the energy of the system. The larger the individual free surface energies, the larger the energetic gain upon the formation of an interface.

Above, we have related the temperature sensitivity of the surface energy κ to the surface entropy *S*_s_. For most liquids, the value κ is negative, owing to an increase in entropy at higher temperatures. This entropy increase can be rationalized by decreasing the coordination number at a liquid surface at higher temperatures. However, a few exceptions have been observed to take positive values. For pure silver, a positive temperature sensitivity has been observed in the temperature range between 1200 and 1500 K, which was associated with oxygen [10]. In this case, oxygen acts as a surface-active element that leads to chemical segregations at the surface. A similar phenomenon was reported for Ga–Bi liquid alloys, in which case a positive temperature sensitivity κ has been observed to increase with the bismuth content. The surface enrichment also explained this result in bismuth, which exhibits a lower surface tension than gallium. Our XPS results also hint at an enrichment in tin and indium at the surface of the Ga–In–Sn eutectic alloy. The surface tension of tin and indium at their melting point is 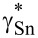 = 689 mN/m and 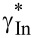 = 562 mN/m, while for pure gallium, the literature reports 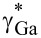 = 713 mN/m [32]. Thus, negative κ values can be explained based on chemical surface segregations. In this line, we suggest that the effect of tip chemistry on κ can be rationalized based on the solubility or reaction of the tip material with the metallic melt. We have already explained the high interfacial energy between SiO*_x_* and the Ga–In–Sn eutectic melt based on the reaction of oxygen atoms from SiO*_x_* with gallium atoms. This reaction is likely to be thermally activated, which would also explain the increase of the interfacial energy with the temperature based on the growth of an interfacial oxide layer. We suggest that tip material comes into solution in the Ga–In–Sn eutectic melt for the gold and platinum silicide tips. Though not verified here, we speculate that the partial and thermally activated solubility ot he tip materials in our metallic melt would form a segregation layer and increase the interfacial tension.

## Conclusion

We have investigated the effect of temperature and chemistry on the interfacial energy between nanoscopic asperities of SiO*_x_*, Au, and PtSi, and a Ga–In–Sn eutectic melt by atomic force spectroscopy. We find that the interfacial energy with Ga–In–Sn eutectic melt is a factor two to eight smaller than its surface tension for all asperities. We find that the interfacial energy is influenced by oxidation of the melt at the SiO*_x_*–liquid metal alloy interface, which results in the largest interfacial energy measured in this work. In the case of gold and platinum silicide, the interfacial energy decreases in proportion to the surface energy of the tip material. Moreover, we observe a positive thermal sensitivity of the interfacial energy, which we explain based on chemical segregation at the interface with the Ga–In–Sn eutectic melt. Beyond the importance of our results for a comprehensive understanding of the physical chemistry of metallic melt interfaces, these results are relevant for designing a microfluidic system with metallic liquids governed by interfacial effects with the channel material.
